# Critical methodological review of 1-year survival outcomes in the intensive care unit

**DOI:** 10.1590/1806-9282.20241504

**Published:** 2025-03-31

**Authors:** Ömerul Faruk Aydın

**Affiliations:** 1 Istanbul Yeni Yüzyıl University, Faculty of Medicine, Department of Emergency Medicine – İstanbul, Turkey.

Dear Editor,

I have read with great interest the article titled "One-year survival after admission in the intensive care unit: a retrospective cohort study," authored by Barisich et al^1^. The manuscript provides valuable insights into intensive care unit (ICU) survival, particularly with a 1-year follow-up period. However, I would like to point out some methodological concerns that may have impacted the findings of the study.

The exclusion of patients who were readmitted or transferred to other hospitals may introduce selection bias^2^. These individuals might have had different clinical outcomes compared to the included cohort, leading to an overestimation of survival rates. This exclusion could compromise the external validity and generalizability of the findings.

Additionally, the study utilizes univariate logistic regression and Cox regression models but does not explore potential interaction terms between variables such as age and APACHE II scores^3^. Interaction effects could significantly influence survival outcomes and should be considered to provide a more comprehensive understanding. Moreover, the lack of a multivariate approach to assess the combined effect of variables such as mechanical ventilation, comorbidities, and severity scores might have oversimplified the conclusions.

The role of mechanical ventilation (MV) was not fully explored in the analysis^4^. While the authors reported that MV did not significantly impact survival, this factor could behave differently when adjusted for other covariates. Furthermore, there is insufficient detail regarding the duration and complications of MV, such as ventilator-associated pneumonia, which could have influenced long-term outcomes.

Although the study appropriately tracks 1-year survival, it fails to address other critical post-ICU outcomes, such as quality of life and functional status^5^. The absence of these data limits the study's ability to provide a holistic assessment of ICU outcomes. Studies on ICU survival typically benefit from the inclusion of such metrics, which help clinicians better understand the full scope of recovery or decline after critical illness.

The manuscript briefly mentions that 16% of the cohort included COVID-19 patients, but it does not provide specific subgroup analyses^6^. Given the known severity of COVID-19 in ICU settings, a more detailed analysis of this group would add depth to the findings.

In conclusion, the authors have made a significant contribution by highlighting ICU survival rates in a Chilean context. However, addressing these methodological issues would strengthen the robustness and applicability of the study.
